# Potential errors in conventional DOT measurement techniques in shake flasks and verification using a rotating flexitube optical sensor

**DOI:** 10.1186/1472-6750-11-49

**Published:** 2011-05-11

**Authors:** Sven Hansen, Frank Kensy, Andreas Käser, Jochen Büchs

**Affiliations:** 1 AVT. Biochemical Engineering, RWTH Aachen University, Worringerweg 1, 52074 Aachen, Germany

## Abstract

**Background:**

Dissolved oxygen tension (DOT) is an important parameter for evaluating a bioprocess. Conventional means to measure DOT in shake flasks using fixed Clark-type electrodes immersed in the bulk liquid are problematic, because they inherently alter the hydrodynamics of the systems. Other approaches to measure DOT that apply fluorescing sensor spots fixed at the inside wall of a shake flask are also suboptimal. At low filling volumes for cultivating microorganisms with a high oxygen demand, the measured DOT signal may be erroneous. Here, the sensor spot is sometimes exposed to gas in the head space of the flask. Merely repositioning the sensor spot elsewhere in the flask does not address this problem, since there is no location in the shake flask that is always covered by the rotating bulk liquid. Thus, the aim of this prospective study is first, to verify the systemic error of Clark-type electrodes for measuring DOT in shake flasks. The second principle aim is to use the newly built "flexitube optical sensor" to verify potential errors in conventional optical DOT measurements based on fixed sensor spots.

**Results:**

With the Clark-type electrode, the maximum oxygen transfer capacity in shake flasks rose compared to that of an analogous system without an electrode. This proves changed hydrodynamics in the system with the Clark-type electrode. Furthermore, regarding the sensor spot experiments under oxygen-limited conditions where the DOT value ought to approach zero, the acquired signals were clearly above zero. This implies that the sensor spot is influenced by oxygen present in the headspace and not only by oxygen in the bulk liquid.

**Conclusions:**

The Clark-type electrode is unsuitable for measuring DOT. Moreover, the newly built rotating flexitube optical sensor is useful to verify potential errors of conventional optical DOT measurement techniques applying fixed sensor spots.

## Background

Shake flasks are widely used in biotechnological research and industry [1-3]. For gaining a better understanding and control of shake flask cultivations, various methods for online monitoring of process parameters in shake flask cultivations have been developed in recent years. Relevant parameters to quantify are oxygen transfer rate (OTR), carbon dioxide transfer rate (CTR), respiratory quotient (RQ), pH and dissolved oxygen tension (DOT).

Anderlei et al. presented the Respiration Activity MOnitoring System (RAMOS) which allows for the online-determination of OTR, CTR and RQ in shake flasks [4,5]. Moreover, Weuster-Botz et al. [6] measured pH-values in shake flasks using standard autoclavable pH-probes that are immersed into the bulk liquid. More recently, fluorescence optodes were used to measure the pH optically and, thus, non-invasively [7,8]. Scheidle et al. [9] combined an optical pH-measurement and the RAMOS principle in one device.

For measuring DOT in shake flasks, several techniques have been introduced so far. Hirose et al. [10] and McDaniel and Bailey [11] measured DOT by using polarographic oxygen sensors. The company teleBITcom (teleBITcom gmbh, Teltow, Germany) developed and commercialized the product series SENBIT, which also enables the measurement of DOT by using a Clark-electrode immersed in the liquid phase [12,13]. However, the possibility of baffling effects by these electrodes, which might significantly change the liquid hydrodynamics, is also mentioned in the literature [14].

Besides the use of a conventional electrode for determining DOT, other methods employing optical sensors have been developed. These sensors are based on the effect of dynamic quenching of luminescence [15]. Tolosa et al. [16] as well as Gupta and Rao [17] fixed an oxygen-sensitive optical sensor spot on the inner flat part of the flask bottom - a technique commercialized by Fluorometrix Corp. (Stow, MA, USA). With this method, the course of DOT during the fermentation of yeast and *E. coli *could be monitored online [16,17]. Wittmann et al. [18] used an optical sensor spot commercialized by PreSens (Sensor, PreSens GmbH, Regensburg, Germany), which is also immobilized on the flat part of the flask bottom. The DOT was successfully measured in cultivations of *Corynebacterium glutamicum *[18,19]. Moreover, Schneider et al. [20] developed an optical device for monitoring DOT and pH in shake flasks. Optical DOT monitoring was also demonstrated for microtiter plates [21,22].

The aforementioned methods are very useful for cultivations where low shaking frequencies and high filling volumes are applied, e.g. for mammalian cells. At these operating conditions, the optical sensor spots are permanently immersed in the bulk liquid. In contrast, at operating conditions used, for example, for bacteria and yeast with high oxygen demand (high shaking frequency, low filling volume), there is no location in the shake flask which is permanently covered by bulk liquid [23]. Due to the shaking motion, the bulk liquid forms a compact liquid body rotating in the flask and thereby distributes a liquid film at the flask wall. This liquid film significantly contributes to the gas-liquid mass transfer [24,25]. The aim of this prospective study is to verify these hypothesized errors of the aforementioned conventional methods on hand of a newly built "flexitube optical sensor".

## Results and Discussion

### Sulfite oxidation experiment

**Figure 1:**
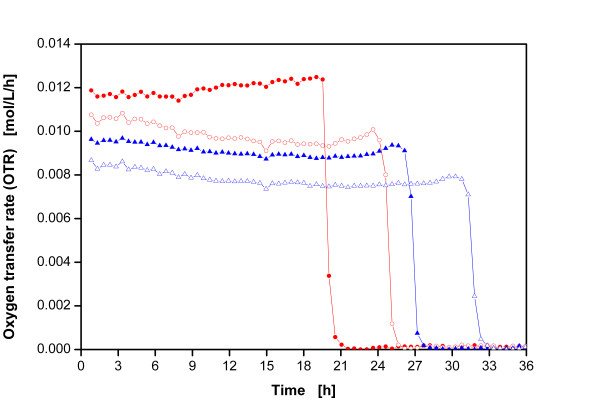
**Oxygen transfer rate during sulfite oxidation in shake flasks with and without an electrode immersed into the rotating bulk fluid; experimental conditions: 0.5 M Na_2_SO_3_, 0.012 M phosphate buffer, 10^-7 ^CoSO_4_, initial pH = 8, T = 25°C, shaking diameter d_0 _= 50 mm, shaking frequency n = 150 rpm; (closed circle) filling volume V_L _= 30 ml, with electrode, (open circle) filling volume V_L _= 30 ml, without electrode, (closed triangle) filling volume V_L _= 40 ml, with electrode, (open triangle) filling volume V_L _= 40 ml, without electrode**.

The maximum oxygen transfer capacities of shake flask systems with and without Clark-type electrodes were measured on hand of sulfite oxidation to check possible changes in the hydrodynamics of the various systems. As Figure 1 illustrates, the shake flasks with an electrode show maximum oxygen transfer capacities of 0.012 mol/L/h and 0.009 mol/L/h for filling volumes (V_L_) of 30 ml and 40 ml, respectively. By contrast, the measurements in the flasks without an electrode depict considerably lower maximum oxygen transfer capacities of 0.01 mol/L/h and 0.008 mol/L/h for filling volumes (V_L_) of 30 ml and 40 ml, respectively. The areas below all four curves are equal, reflecting a total oxygen consumption of 0.24 mol/L. This corresponds to the stoichiometric amount of oxygen consumed by a 0.5 mol/L Na_2_SO_3 _solution [26].

The results indicate that a Clark-type electrode indeed acts as a baffle, thereby increasing the whole oxygen transfer of the system. Consequently, conventional Clark-type electrodes considerably change the hydrodynamics in the flask and are not recommended as measurement devices as the flask with electrodes do not represent the culture conditions in the conventional reference flasks.

### Optical DOT measurements with a fixed sensor spot

**Figure 2:**
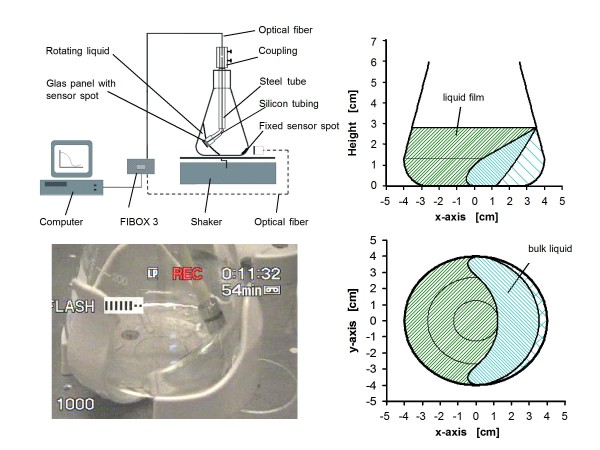
**Experimental setup of dissolved oxygen tension (DOT) measurement with rotating flexitube optical sensor system and with sensor spot fixed to the inside flask wall (dashed line) **(A); photo of rotating flexitube optical sensor in shake flask (B); computed liquid distribution in a 250 ml shake flask according to Maier and Büchs [24], shaking frequency n = 150 rpm, shaking diameter d_0 _= 50 mm, filling volume V_L _= 25 ml, side view (C) and top view (D).

To avoid altered hydrodynamics of the system, an oxygen-sensitive fluorescent sensor spot was investigated to measure DOT in a fermentation with *E. coli *BL21 pET28a. Furtermore, OTR was measured by using a RAMOS device. As Figure 2A shows, the sensor spot was fixed in the inside corner of the flask and not centered on the bottom of the flask as proposed in the literature [16-18,20]. Please note that the inside corner is the optimal position for the sensor spot. According to Büchs et al. [23], the rotating bulk liquid has the longest contact time to the shake flask wall at this corner position.

**Figure 3:**
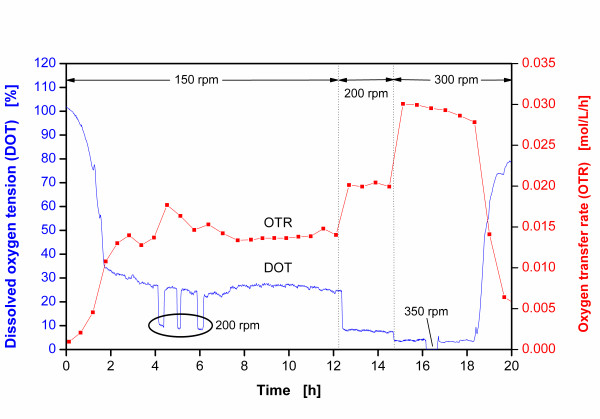
**Online signals of dissolved oxygen tension (DOT) and oxygen transfer rate (OTR) in a cultivation of *E. coli *BL21 pET28a in TB medium at different shaking frequencies**. DOT was measured with sensor spot fixed to the inside flask wall (refer to Fig. 2A); experimental conditions: temperature T = 37°C, shaking diameter d_0 _= 50 mm, filling volume V_L _= 25 ml, initial optical density OD_α _= 0.3.

Figure 3 illustrates the various OTR and DOT values measured during this fermentation, whereby the shaking frequencies were altered in the range of 150-350 rpm. At 150 rpm, the OTR increased exponentially during the first 2-3 h after inoculation, while the DOT values dropped correspondingly. For all shaking frequencies, the constant OTR values from 3 h fermentation onwards indicate that the cultivation becomes oxygen-limited. This is clearly pointed out by the plateaus in the OTR curve [4,5]. This oxygen limitation is also substantiated by a sharp increase in the OTR upon increasing the shaking frequency to 200 rpm and 300 rpm, respectively. This increase in the shaking frequencies results in a better gas-liquid mass transfer. Furthermore, oxygen limitations of *E. coli *cultures in shake flasks with TB medium have already been detected under even more favorable cultivation conditions (higher shaking frequencies and lower filling volumes) [27]. The DOT is expected to approach 0% at all applied shaking frequencies, because the Monod constant of bacteria is very small [28].

By using the sensor spot, DOT values ranging from 23-28% were found at 3 to 12 h after inoculation at a shaking frequency n = 150 rpm. Temporarily raising the shaking frequency to 200 rpm during the period of 4 to 6 h after inoculation and constantly raising it to 200 rpm during the period of 12 to 15 h after inoculation, both lead to a DOT value of ca. 9%. Even again increasing the shaking frequency to 300 rpm between 15 to 18 h after inoculation results in a DOT value of approximately 5%. After temporarily raising the shaking frequency to 350 rpm for just 30 min at around 16 h after inoculation produced a DOT value approaching 0%. Only this value measured at 350 rpm concurs with the oxygen limitation proven by the OTR measurements using RAMOS. Even though the DOT values under the proven oxygen limitation of the system ought to approach 0%, they do not. Instead, they show significantly higher values at all applied shaking frequencies below 350 rpm.

Thus, this deviation in actual DOT values implies that the sensor spot is not always in contact with the bulk liquid during the flask rotation. According to Büchs et al. and Maier et al. [23-25], at high shaking frequencies and low filling volumes there is no location in the shake flask that is always covered by the rotating bulk liquid. This fact is presented in Figure 2C-D for the conditions cited in Figure 3 for the first 12 hours after inoculation. Since the sensor spot in the prospective study has been fixed at the optimal corner location of the flask, merely repositioning it elsewhere in the flask does not improve the DOT measurement. The sensor spot may possibly register the oxygen concentration of the headspace as well as that of the thin liquid film caused by the rotating bulk liquid [24]. The liquid film typically has a higher mean DOT value than that of the bulk liquid, because the film is enriched with oxygen from the headspace. The effect of the liquid film on the DOT value recorded by the fixed sensor spot becomes apparent by considering that, at lower shaking frequencies, a greater surface area of the inside flask wall is wetted by the bulk liquid, as was predicted by Büchs et al. and Maier et al. [23-25]. Taking into account only the bulk liquid and the gas headspace would lead to lower DOT values at lower shaking frequencies, contrary to Figure 3.

Consequently, the liquid film significantly influences the DOT signal generated by the fixed sensor spot. The higher the shaking frequency, the less oxygen can be enriched in the liquid film. In this case, the film has less time to drain down the flask wall within one rotating cycle. Thus, this effect at higher shaking frequencies, leads to DOT signals closer to the real DOT values indicated by the OTR values measured under oxygen-limited conditions. Moreover, as seen in Figure 2C-D, the center of the flask bottom has no contact with the liquid at all. Therefore, under these conditions it would be a big error to place the sensor spot in the center of the flask bottom.

It should be pointed out that the unrealistically high DOT values do not reflect the low oxygen concentration constantly experienced by the microorganisms under oxygen-limited conditions as these microorganisms rotate with the bulk liquid. This oxygen limitation, in turn, may result in anaerobic byproducts which would not be reflected by the higher DOT measurements with the fixed sensor spot. This clearly proves that an optical DOT measurement with a fixed sensor spot is not reliable for every possible cultivation condition. This method is merely reliable for cultivations where high filling volumes and low shaking frequencies are applied, e.g., for the cultivation of mammalian cells. Triggering the reading of the sensor signal to coincide at that time point when the bulk liquid is actually contacted is not feasible, because the sensor spot has a lag time of approximately 6 seconds [29]. This lag period is much higher than the duration of one rotating cycle.

### Optical DOT measurements with rotating flexitube optical sensor

**Figure 4:**
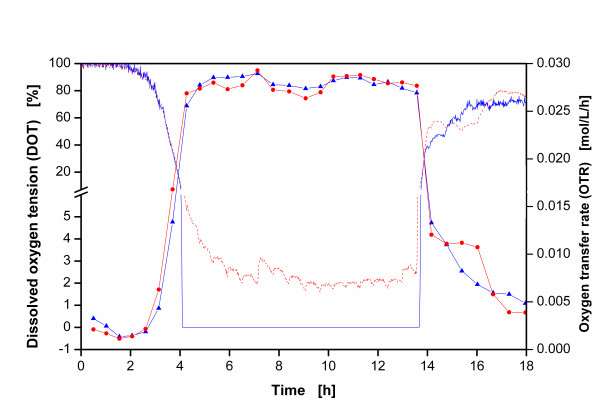
**Online signals of dissolved oxygen tension (DOT) and oxygen transfer rate (OTR) in a cultivation of *E. coli *BL21 pET28a in TB medium**; experimental conditions: temperature T = 37°C, shaking diameter d_0 _= 25 mm, shaking frequency n = 300 rpm, filling volume V_L _= 25 ml, initial optical density OD_α _= 0.3; (dashed line) DOT measured with a sensor spot fixed to the inside flask wall, (full line) DOT measured with rotating flexitube optical sensor system according to Fig. 2, (circle) OTR in flask without flexitube optical sensor, (triangle) OTR in flask with flexitube optical sensor.

In order to further verify potential errors in optical DOT measurement techniques, DOT experiments were set up with the newly constructed rotating flexitube optical sensor. Figure 4 illustrates the results of the OTR and DOT measurements in two cultivations of *E. coli *BL21 ET28a in shake flask either having a fixed sensor spot in the inside corner of the flask or the new rotating flexitube optical sensor as is described in "Methods". It should be noted that the OTR values for both experimental setups are identical. After the exponential growth phase, the OTR values reach a plateau at about 0.028 mol/L/h between 4 and 14 h after inoculation. This plateau indicates an oxygen limitation during this period [4]. Afterwards, the OTR curve plummets due to the depletion of the C-source.

The measured DOT curves for both systems (with fixed sensor spot and with the rotating flexitube optical sensor) are identical during the first 4 hours after inoculation. Afterwards, the DOT curve acquired with the fixed sensor spot drops to a DOT value of ca. 2-4% between 4 h and 14 h after inoculation. Thereafter, this curve rapidly increases until the end of the fermentation. The regular fluctuations of this curve correspond to the typical gassing cycles of the RAMOS device [4,5]. By contrast, the rotating flexitube optical sensor system records a signal of 0% DOT during the whole time of oxygen limitation. Consequently, the former DOT curve (with fixed sensor spot) shows an error of 2-4%. This error is attributed to the fixed sensor spot not always being in contact with the bulk liquid but rather with gas in the head space of the flask or the liquid film forming at the flask wall. Even though the absolute DOT values of the systems may vary within a minute, the fact that an oxygen limitation is not detected using the fixed sensor spot is very significant. This erroneous reading may lead to a complete misinterpretation of the results.

Since the DOT signal of the rotating flexitube optical sensor system agrees with the oxygen limitation recorded by the RAMOS device, this new sensor system is more accurate than the fixed sensor spot. Due to the centripetal forces during shaking, the flexitube optical sensor rotates freely and in sync to the bulk liquid. Therefore, the sensor is always in contact with the bulk liquid and neither registers oxygen from the headspace nor from the liquid film.

Even though the optode at the tip of the rotating flexitube optical sensor blocks the mass transfer of oxygen at this point, this blockage is insignificant. To quantify this blocked fraction, the area of the tip of the rotating optode (0.79 cm^2^) is compared with the total mass transfer area in shake flasks under the investigated conditions. This total mass transfer area can be calculated according to Büchs et al [23]. Corresponding to the operating conditions depicted in Figure 4, the total gas-liquid mass transfer area is 103.19 cm^2^. Compared to this area, the rotating flexitube optical sensor system merely blocks 0.77% of the total mass transfer area and, thus, can be disregarded. Moreover, as Figure 4 shows, the measured OTR values of both systems show exactly the same OTR values. This signifies that the rotating flexitube optical sensor system neither affects the hydrodynamics nor the oxygen transfer into the liquid.

## Conclusions

Conventional means to measure DOT in shake flasks may be erroneous. Since Clark-type electrodes act like a baffle, they inherently alter the hydrodynamics of the system and raise the maximum oxygen transfer capacity. Furthermore, optical DOT measurement techniques applying fixed sensor spots may lead to erroneous results under fermentation conditions for microorganisms requiring a high oxygen demand. Under low filling volumes, the fixed sensor spot may register gas in the head space of the flask and oxygen concentrations in the liquid film forming along the glass wall of the rotating flask. Thus, the DOT signals generated under these conditions are incorrect. If at high shaking frequency and low filling volume no constant contact of the fixed sensor spot with the rotating bulk liquid can be ensured, it should be recognized that an oxygen limitation and a DOT close to zero will not be correctly indicated by the signal. Limiting oxygen concentrations will be indicated by horizontal signals over time at levels of up to 30% air saturation. The corresponding values can be estimated from Figure 3.

However, the newly built rotating flexitube optical sensor is always in contact with the bulk liquid. Thus, it is useful to verify potential errors in the conventional optical DOT measurement technique that uses fixed sensor spots. Nonetheless, this rotating flexitube optical sensor requires a somewhat complicated setup and is not durable as a fixed sensor spot system. Even so, this new technique is useful to verify potential errors in conventional optical DOT measurements.

## Methods

### Sulfite oxidation experiment

**Figure 5:**
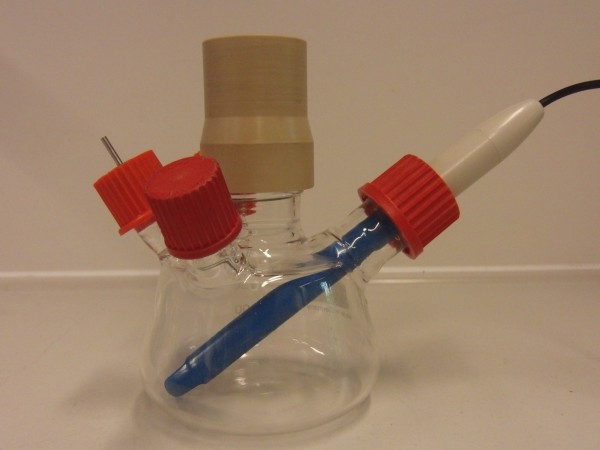
**Modified 250 mL Erlenmeyer flasks with inserted electrode for sulfite oxidation experiments in RAMOS**.

The sulfite oxidation experiment, as described by Hermann et al. [26], was performed in modified 250 mL Erlenmeyer flasks at T = 25°C with filling volumes of both V_L _= 30 ml and V_L _= 40 ml using the Respiration Activity MOnitoring System (RAMOS). The sulfite solution consists of 0.5 mol/L Na_2_SO_3_, 0.012 mol/L Na_2_HPO_4_/NaH_2_PO_4 _and 10^-7 ^mol/L CoSO_4_. The pH-value was adjusted to 8 with sulfuric acid. For each filling volume, a flask with an electrode and a flask without an electrode were respectively used. The shake flasks with an electrode each had an additional melted-on glass adaptor into which an electrode with a diameter of 12 mm was inserted as shown in Figure 5. The shaking frequency was n = 150 rpm, and the shaking diameter was d_0 _= 50 mm. part of the flask, as proposed in literature [16-18,20]. In addition, an optical fiber reading unit was fixed at the outside of the shake flask and was connected to the FIBOX 3 device.

### Optical DOT measurements with rotating flexitube optical sensor

The rotating flexitube optical sensor consists of an adjustable stainless steel tube extending 90 mm from the upper flask neck into the flask. It is connected to a flexible silicon tubing (Ismatec, Pharmed Ismaprene, inner diameter: 4.8 mm, outer diameter: 8 mm, length: 55 mm) at its lower end (Figure 2A-B). The steel tube is mounted on a modified 250 ml Erlenmeyer flask using a flange and a compatible screw cap. At the end of the silicon tubing a glass panel (diameter: 10 mm) is fixed which incorporates an oxygen-sensitive fluorescent sensor spot at the outer side of the glass panel. Inside the steel tube and the silicon tubing is an optical fiber within an inner tubing that leads to the FIBOX 3 device.

### Respiration Activity Monitoring System (RAMOS)

The OTR values were measured using a self-made RAMOS device in modified 250 mL Erlenmeyer flasks as introduced by Anderlei and Büchs [4] and Anderlei et al. [5]. These flasks ensure the same hydrodynamic conditions and headspace gas concentrations as are found in regular Erlenmeyer flasks with cotton plugs [5]. Commercial versions are available from Kuehner AG, Birsfelden, Switzerland and Hitech Zang, Herzogenrath, Germany.

## Authors' contributions

SH drafted the manuscript. FK and AK performed the experiments. Having conceived the study, JB helped to design, coordinate and write the manuscript. All authors read and approved the final manuscript.
